# Identification of the α2 chain of interleukin‐13 receptor as a potential biomarker for predicting castration resistance of prostate cancer using patient‐derived xenograft models

**DOI:** 10.1002/cnr2.1701

**Published:** 2022-08-09

**Authors:** Takahiro Nagai, Naoki Terada, Masato Fujii, Yasuhisa Nagata, Kozue Nakahara, Shoichiro Mukai, Kosuke Okasho, Yuki Kamiyama, Shusuke Akamatsu, Takashi Kobayashi, Kei Iida, Masatsugu Denawa, Masatoshi Hagiwara, Takahiro Inoue, Osamu Ogawa, Toshiyuki Kamoto

**Affiliations:** ^1^ Department of Urology Miyazaki University Graduate School of Medicine Miyazaki Japan; ^2^ Department of Urology Kyoto University Graduate School of Medicine Kyoto Japan; ^3^ Medical Research Support Center, Graduate School of Medicine Kyoto University Kyoto Japan; ^4^ Department of Anatomy and Developmental Biology, Graduate School of Medicine Kyoto University Kyoto Japan; ^5^ Department of Nephro‐Urologic Surgery and Andrology Mie University Graduate School of Medicine Tsu Japan

**Keywords:** biomarker, castration resistance, Interleukin‐13 receptor α2, patient‐derived xenograft, prostate cancer

## Abstract

**Background:**

Several treatment strategies use upfront chemotherapy or androgen receptor axis‐targeting therapies for metastatic prostate cancer. However, there are no useful biomarkers for selecting appropriate patients who urgently require these treatments.

**Methods:**

Novel patient‐derived xenograft (PDX) castration‐sensitive and ‐resistant models were established and gene expression patterns were comprehensively compared. The function of a gene highly expressed in the castration‐resistant models was evaluated by its overexpression in LNCaP prostate cancer cells. Protein expression in the tumors and serum of patients was examined by immunohistochemistry and ELISA, and correlations with castration resistance were analyzed.

**Results:**

Expression of the α2 chain of interleukin‐13 receptor (IL13Rα2) was higher in castration‐resistant PDX tumors. LNCaP cells overexpressing IL13Rα2 acquired castration resistance in vitro and in vivo. In tissue samples, IL13Rα2 expression levels were significantly associated with castration‐resistant progression (*p* < 0.05). In serum samples, IL13Rα2 levels could be measured in 5 of 28 (18%) castration‐resistant prostate cancer patients.

**Conclusion:**

IL13Rα2 was highly expressed in castration‐resistant prostate cancer PDX models and was associated with the castration resistance of prostate cancer cells. It might be a potential tissue and serum biomarker for predicting castration resistance in prostate cancer patients.

## 1 INTRODUCTION

Prostate cancer is the second leading cause of cancer‐related death in men in the Western countries^1^ and its incidence in Japan is increasing rapidly.^2^ Androgen deprivation therapy (ADT) is an effective initial treatment for patients with advanced disease harboring distant metastasis. However, these lesions acquire therapeutic resistance within several years and progress to castration‐resistant prostate cancer (CRPC). To improve the prognosis of patients by prolonging time to CRPC progression, upfront docetaxel^3^ or androgen receptor axis‐targeting agents (ARATs)^4^, ^5^, ^6^ combined with ADT have become the standard treatment for metastatic hormone‐naïve prostate cancer (HNPC). However, ADT monotherapy is effective for a long duration in some HNPC patients for whom upfront therapies are not needed. At present, there are no useful biomarkers for selecting appropriate patients who really need these upfront therapies.

We previously established various novel patient‐derived xenograft (PDX) models named KUCaPs through the direct inoculation of tumor tissues obtained from CRPC patients into immunodeficient mice.^7^, ^8^, ^9^ They all expressed androgen receptor (AR) and prostate‐specific antigen (PSA). However, castration sensitivity differed among the models. They were grouped into castration‐sensitive and ‐resistant models based on the sequential tumor volume after castration of the mice. By comparing the gene expression of these tumors, we explored biomarkers for predicting the castration resistance of prostate cancer and found that the α2 chain of interleukin‐13 receptor (IL13Rα2) was highly expressed in castration‐resistant models. Several studies found that IL13Rα2 was a therapeutic target in cancer cells^10^, ^11^, ^12^, ^13^ and in prostate cancer.^14^ In this study, we evaluated the function of IL13Rα2 associated with the castration resistance of prostate cancer and explored its clinical utility as a tissue and serum biomarker for predicting castration resistance in prostate cancer patients.

## 2 MATERIALS AND METHODS

### 2.1 Generation of PDX models

Clinical materials were used after informed consent was obtained, in accordance with protocols approved by the institutional review boards at Kyoto University Hospital (approval number: G52). Tumor tissues were obtained from CRPC patients, when local recurrent tumors were resected transurethrally for the treatment of urinary symptom or bone metastatic tumors were extracted at the surgical treatment for spinal cord compression. Tumor tissues were minced into 20–30 mm^3^ pieces and transplanted subcutaneously into 5‐week‐old male nude mice (Charles River Japan, Yokohama, Japan) with 50 μl Matrigel (Becton Dickinson, Franklin Lakes, New Jersey, USA) injected around the implant. The PDX models were named KUCaPs^7^, ^8^, ^9^ and were established approximately 6–10 months after the first inoculation. The KUCaP tumors were extracted and transplanted into several mice without Matrigel. Ninety percent of the tumor was serially transplantable.

### 2.2 Cell lines

LNCaP and PC3 cells were purchased from the American Type Culture Collection (Manassas, Virginia, USA) and cultured under typical conditions in RPMI 1640 (Invitrogen) supplemented with 10% fetal bovine serum (FBS). For androgen‐depleted conditions, cells were cultured in phenol red‐free RPMI 1640 (Invitrogen) supplemented with 10% charcoal‐stripped fetal bovine serum (CSFBS) (Hyclone). Cell numbers were counted using a Countess II (Invitrogen).

### 2.3 Tissue sampling and whole transcriptome analysis

Mice bearing KUCaP tumors were castrated and the sequential changes in tumor volume were analyzed as the previously reported.^7^ The mice without castration were killed when the tumor size was approximately 500 mm^3^. From each collected tumor tissue, total RNA was extracted using the RNeasy Midi Kit (Qiagen, Valencia, CA, USA). The quality of isolated total RNA was examined using the Agilent RNA 6000 Nano Kit and the Agilent 2100 Bioanalyzer, and RNA integrity numbers (RIN) ranged 6.30–9.83. Then, 10 μg total RNA with 2 μl diluted Ambion ERCC RNA Spike‐in Mix (1:10 dilution; Life Technologies) was treated with Dynabeads mRNA DIRECT™ Microkit (Life Technologies) to prepare polyA‐selected messenger RNA. RNA libraries were then prepared using the Ion Total RNA‐Seq Kit for the AB Library Builder™ System, and the AB Library Builder™ System (Life Technologies). Prepared libraries were purified with Agencourt AMPure XP (Beckman Coulter). High‐throughput sequencing (HTS) was performed using the Ion Proton System. Experiments were performed in biological triplicate.

### 2.4 Bioinformatics analysis of RNA‐Seq

For quality control (QC), sequence read lengths <75 and average Phred scores <17 were discarded from the study. Sequence reads mapped to the murine leukemia virus (KU324806.1) or RNA elements (rRNA/tRNA/snRNA/snoRNA/pseudo genes) of humans or mice, respectively, were checked with Bowtie2 (ver. 2.1.0)^15^ and discarded. To prepare the sequence information for RNA elements, we used Genbank, UCSC Genome Browser, and Ensembl.^16^, ^17^, ^18^ Between 14.4%–38.2% of sequence reads were discarded during this process. After QC, the passed sequence reads were mapped to the mixed database of human (hg19) and mouse (mm10) genomes with STAR aligner using Encode options.^19^ The remaining 38.9%–69.8% QC‐passed reads were mapped to either of the genomes. Uniquely mapped reads were used for the following analyses to trace species‐specific expression of the genes. Fragments per kilobase million (FPKM) were calculated for the human genes (Refseq, GRCh37.p5)^20^ with the method described previously,^21^, ^22^ then transferred to transcripts per million (TPM) values.

### 2.5 Real‐time PCR

Tissues were homogenized by a μT‐1(TAITEC, Nagoya, Japan) before extracting RNA. Total RNA of cells and tissue was extracted using the PureLink RNA Mini Kit (Thermo Fisher, Waltham, MA, USA). cDNA was synthesized from total RNA using PrimeScript Reverse Transcriptase (Takara, Shiga, Japan) and random primers. Real‐time RT‐PCR analyses were performed with a Thermal Cycler Dice Real‐Time System II (Takara, Shiga, Japan). Reaction mixture (25 μl) containing 2 μl cDNA template, 1 μl each of sense and anti‐sense primers, and 1 × SYBR Premix Ex Taq II (Takara, Shiga, Japan) were amplified as follows: hold at 95°C for 30 s, then 40 cycles at 95°C for 5 s, 60°C for 30 s, followed by a final dissociation stage (95°C for 15 s, 60°C for 30 s, and 95°C for 15 s). Glyceraldehyde‐3‐phosphate dehydrogenase (*GAPDH*) was used as an internal control. The results were evaluated using the Thermal Cycler Dice Real Time System software program (Takara Bio, Shiga, Japan), and the ΔΔCt algorithm was used to analyze the relative changes in gene expression. The primers were as follows: *GAPDH* forward, 5′‐GCACCGTCAAGGCTGAGAAC‐3′ and reverse, 5′‐TGGTGAAGACGCCAGTGGA‐3′; *IL13RA2* forward, 5′‐TTGCTTGGCTATCGGATGCT‐3′ and reverse, 5′‐GGGTTAACTTTTATCTCGGTGTCTGA‐3′; *AR* forward, 5′‐TCCATTGCCCACCAAAGACT‐3′ and reverse, 5′‐GCAAATCTGGCCTGTCACCT‐3′; *KLK3* forward, 5′‐GGAAATGACCAGGCCAAGAC‐3′ and reverse, 5′‐CAACCCTGGACCTCACACCTA‐3′. All experiments were performed in triplicate.

### 2.6 Transfection

LNCaP cells were infected with lenti‐open reading frame particles encoding *IL13RA2* GFP (Cat #: RC207672L4V) or a non‐targeted control GFP (Cat #: PS100093V) (Origene, Rockville, MD) using 5 μg/ml polybrene (Sigma). Infected cells (LNCaP‐mock and LNCaP‐IL13Ra2) were selected through constant culture in 1 μg/ml puromycin. IL13Ra2 overexpression was confirmed by real‐time PCR and immunoblotting. Efficiency of the transfection was determined by GFP fluorescence microscopy.

### 2.7 Cell proliferation assay

In a 96‐well plate, 3 × 10^3^ cells were plated in 100 μl medium and incubated for the indicated period, after which 20 μl CellTiter 96 Aqueous One Solution (Promega) was added. After an additional 1‐h incubation at 37°C, the absorbance of each well at 490 nm was measured. The cells were incubated in normal and androgen‐depleted medium with or without adding 25 mg/ml of IL13 recombinant protein (R&D Systems) for 72 h, followed by 3‐(4,5‐dimethylthiazol‐2‐yl)‐5‐(3‐carboxymethoxyphenyl)‐2‐(4‐sulfophenyl)‐2H‐tetrazolium (MTS) assay. All experiments were performed three times in triplicate.

### 2.8 Cell‐derived xenograft tumor formation and proliferation assay

Cell‐derived xenografts (CDXs) were developed using 5 × 10^6^ LNCaP‐mock and LNCaP‐IL13Ra2 cells. Cells were suspended in 200 μl media and subcutaneously injected into both flanks of 6‐week‐old nude mice. For the mice in which tumors were detected, surgical castration was performed 5 weeks after transplantation. Tumor volumes were measured with a caliper using the formula, *a* × *b* ^2^ × 0.52, where a is the largest diameter and *b* is the largest diameter perpendicular to *a*.

### 2.9 Western blotting

Anti‐AR (#3202), PSA (#2475), and α‐tubulin (#2144) antibodies were obtained from Cell Signaling Technology. MAB614 monoclonal antibody against human IL13Rα2 was purchased from R&D Systems (Minneapolis, NM). Western blotting was performed as previously reported.^8^ Cells were washed twice with ice‐cold PBS followed by incubation with the RIPA lysis buffer (Thermo Fisher Scientific) at 4°C. The extracted protein was collected by centrifugation at 12000 rpm for 15 min at 4°C. Protein concentrations were determined by using the bicinchoninic acid method (Takara, Shiga, Japan). Equal amounts of protein were resolved by sodium dodecyl sulfate‐polyacrylamide gel electrophoresis (SDS‐PAGE) and transferred to polyvinylidene difluoride filters. The membrane was blotted with monoclonal antibodies (AR, 1:400; PSA, 1:400; IL13Ra2, 1:200; α‐tubulin, 1:5000) by using the WesternBreeze chromogenic immunodetection system (Thermo Fisher Scientific).

### 2.10 Immunohistochemistry

Immunohistochemical analysis was performed on formalin‐fixed, paraffin‐embedded clinical samples or xenograft tissues. It was conducted by processing sections for antigen retrieval (microwaved in 10 mM citrate buffer, pH 6.0 for 10 min), followed by treatment with 3% H_2_O_2_ in methanol for 10 min and washing in phosphate‐buffered saline (PBS) twice. After blocking in 3% bovine serum albumin and 5% goat serum in phosphate buffered saline for 2 h at room temperature, sections were incubated with primary antibody (anti‐IL13Ra2 1:200; 11 059‐1‐AP; Proteintech, Wuhan, China) for 4 h at 4 °C. Sections were washed in PBS and incubated with EnVision‐labeled polymer reagent (DAKO, Carpinteria, CA, USA) for 30 min at room temperature. Sections were exposed with nickel, cobalt‐3, 3‐diaminobenzidine (Immunopure Metal Enhanced DAB Substrate Kit; Piece, Rockford, IL, USA), and counterstained with hematoxylin.

### 2.11 ELISA

The concentration of IL13Rα2 in serum samples was determined in duplicate with the IL13Rα2 ELISA kit (Thermo Fisher Scientific) in accordance with the manufacturer's recommended protocol.

### 2.12 Tissue and blood samples of patients

Tumor tissue specimens and blood samples were obtained from prostate cancer patients at the Department of Urology, Miyazaki University Hospital with appropriate written informed consent. This study was approved by the Miyazaki University's institutional review board (approval number: O‐0132).

### 2.13 Statistical analysis

Differences between each group were compared by paired *t*‐test. Castration‐resistant disease was defined as progression of serum PSA levels (> 25% relative to the nadir PSA and higher than 2 ng/ml), radiographic finding, and clinical symptom after initial hormonal therapy. The castration‐resistant progression free survival was calculated using the Kaplan–Meier method and log‐rank tests were used to analyze differences between the two groups. Statistical tests were two‐sided and *p*‐values <0.05 were considered statistically significant.

## 3 RESULTS

### 3.1 Differential expression of IL13Rα2 between castration‐sensitive and ‐resistant prostate cancer

**FIGURE 1:**
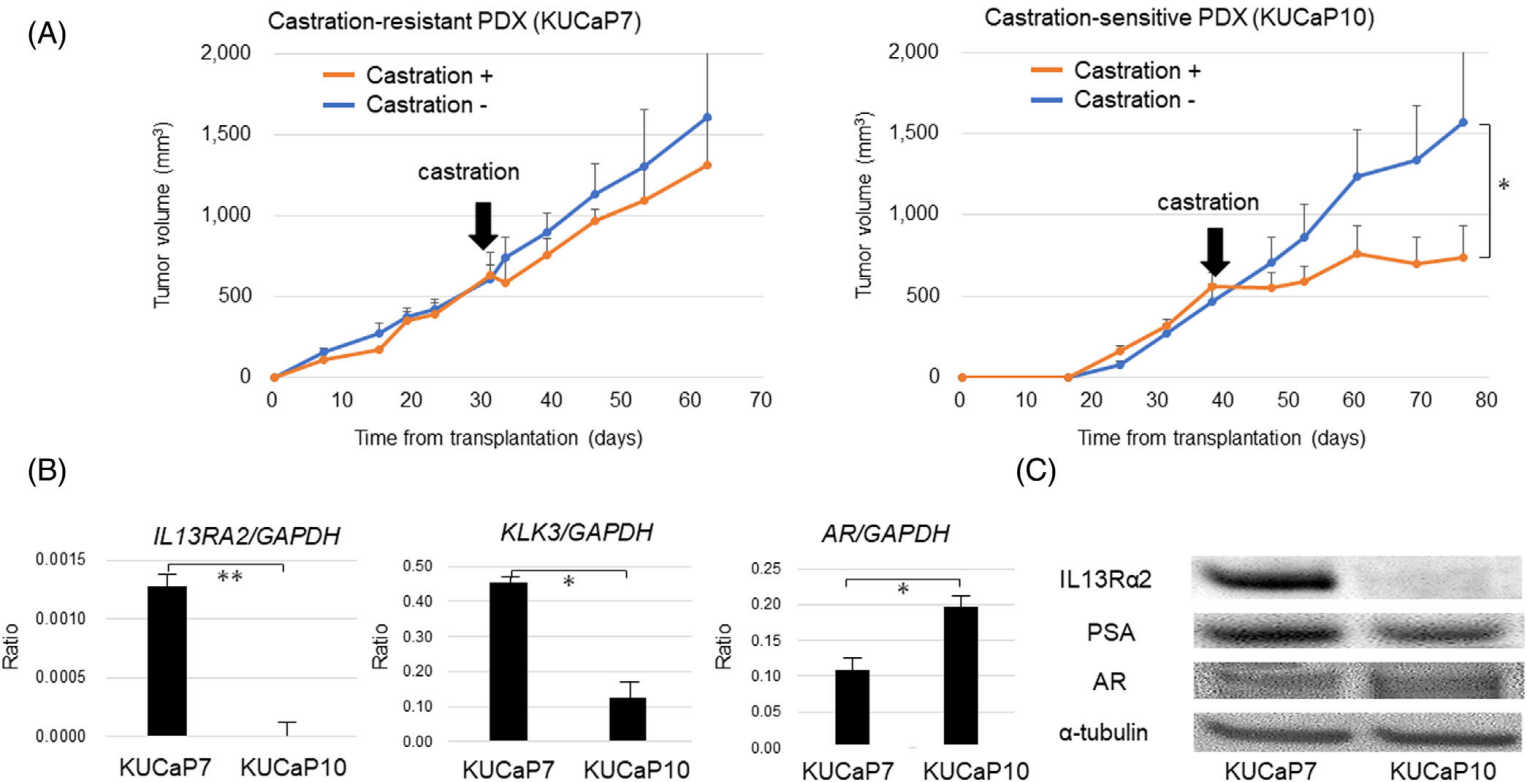
IL13Rα2 expression in castration‐resistant and ‐sensitive PDX tumors.A. Sequential changes in tumor size of a castration‐resistant PDX model (KUCaP7) and a castration‐sensitive PDX model (KUCaP10) in castrated (castration+) and control (castration−) mice (*n* = 5 each) (*p < 0.05). B. IL13RA2, AR, and KLK3 mRNA expression (based on GAPDH) in KUCaP7 and KUCaP10 tumors detected by real‐time PCR (*p < 0.05, **p < 0.005). C. Expression levels of IL13Rα2, AR, and PSA (based on α‐tubulin) in KUCaP7 and KUCaP10 tumors detected by western blotting

We established various PDX models (KUCaPs) using tumor tissues derived from different patients. Of the KUCaPs, KUCaP2 (established from a local recurrent tumor at PSA 3.5 ng/ml with initial Gleason score of 4 + 5, as previously reported),^8^ KUCaP7 (established from a local recurrent tumor at PSA 392 ng/ml with initial Gleason score of 4 + 3), KUCaP9 (established from a bone metastatic tumor at PSA 7.6 ng/ml with initial Gleason score of 5 + 4), and KUCaP10 (established from a bone metastatic tumor at PSA 3.2 ng/ml with initial Gleason score of 4 + 4) were used in this study. The prostate cancer of the patients from whom the PDX model was established were sensitive to the first line ADT. The time to castration‐resistant progression were 30, 26, 12 and 16 months, in patients for KUCaP2, 7, 9, and 10, respectively. To evaluate the sensitivity of these PDX models to castration, mice bearing tumors were castrated and the sequential changes in tumor volume were compared with non‐castrated control mice. There was no significant difference between castrated and control mice in KUCaP7, being classified as a castration‐resistant PDX model. Tumor growth was significantly suppressed by the castration of mice in KUCaP10, which was classified as a castration‐sensitive PDX model (Figure 1A). In the same way, the KUCaP9 model was classified as castration‐resistant and the KUCaP2 model was classified as castration‐sensitive (data not shown). Gene expression patterns between the castration‐resistant PDX models (KUCaP7 and KUCaP9) and the castration‐sensitive PDX models (KUCaP2 and KUCaP10) were comprehensively compared using RNA‐seq. The relative expression levels of *KLK3* calculated in the RNA‐seq data were 11.7 and 7.5, 9.8 and 10.0, and those of *AR* were 5.6 and 4.6, 5.4 and 6.4, in KUCaP 7 and 9 (castration‐resistant PDX models), 2 and 10 (castration‐sensitive PDX models), respectively. The *KLK3* and *AR* mRNA expression were validated by the real‐time PCR in KUCaP7 and KUCaP10 (Figure 1B). Although the mRNA expression levels were different, the protein expression levels of PSA and AR were not significantly different among the KUCaPs (Figure 1C and supplemental Figure 1). These results indicated that the AR expression and signaling activity of the PDX models were not correlated with their castration‐sensitivity. Then, we selected genes for which the expression levels were significantly different (*p* < 0.0005, t‐test) according to the ratio of logarithm transferred (TPM + 1) expression values (base = 2) between castration‐resistant KUCaPs and castration‐sensitive KUCaPs. Among the top 30 genes highly expressed in the castration‐resistant KUCaPs, we focused on IL13Rα2, which is a membranous or secreted protein (Supplemental Table 1). This molecule was previously reported to be associated with cancer progression in various types of cancers^10^, ^11^, ^12^, ^13^, ^14^ and considered as a candidate for tissue or serum biomarkers in prostate cancer.

Both the castration‐resistant (KUCaP7) and castration‐sensitive (KUCaP10) PDX models were shown to express *KLK3* and *AR* by real‐time PCR (Figure 1B) and PSA (encoded by *KLK3* gene) and AR in western blotting (Figure 1C). The differential expression of IL13Rα2 was confirmed by real‐time PCR (Figure 1B) and western blotting (Figure 1C). *IL13RA2* mRNA was also more highly expressed in PC3 cells than in LNCaP cells, indicating that IL13Rα2 was highly expressed in castration‐resistant prostate cancer cells. However, IL13Rα2 protein was not detected in PC3 cells by western blotting (Supplemental Figure 1).

### 3.2 Overexpression of IL13Rα2 in LNCaP cells induced androgen‐independent proliferation and differential response to IL‐13 administration

**FIGURE 2:**
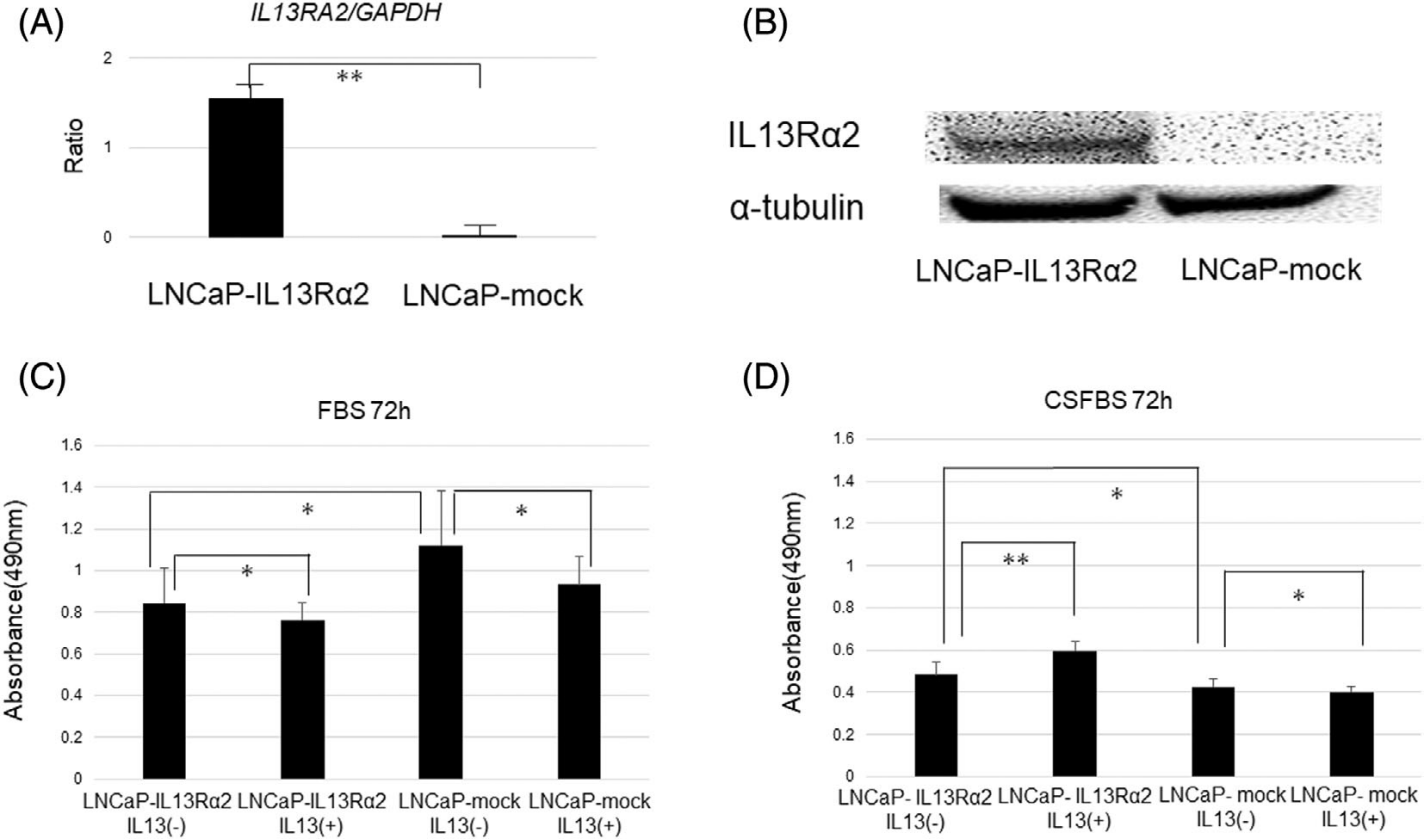
IL13Rα2 overexpression in LNCaP cells enhanced proliferation in androgen‐depleted medium. A. Expression of IL13RA2 mRNA (based on GAPDH) in LNCaP‐mock and LNCaP‐IL13Rα2 by real‐time PCR (**p < 0.005). B. Expression of IL13Rα2 protein (based on α‐tubulin) in LNCaP‐mock and LNCaP‐IL13Rα2 by western blotting. C. Cell numbers of LNCaP‐mock and LNCaP‐IL13Rα2 after 72 h in normal medium (FBS) with or without human IL13 recombinant protein (25 ng/ml) (*p < 0.05). D. Cell numbers of LNCaP‐mock and LNCaP‐IL13Rα2 after 72 h in androgen‐depleted medium (CSFBS) with or without human IL13 recombinant protein (*p < 0.05, **p < 0.005)

LNCaP cells were stably transfected with non‐targeted control GFP‐control (LNCaP‐mock) and IL13Ra2‐GFP (LNCaP‐IL13Rα2). *IL13RA2* mRNA and IL13Rα2 protein were highly expressed in LNCaP‐IL13Rα2 in real‐time PCR and western blotting (Figure 2A, B). LNCaP‐mock and LNCaP‐IL13Rα2 cells were cultured in normal and androgen‐depleted conditions. In normal conditions, the proliferation of LNCaP‐IL13Rα2 was significantly lower than that of LNCaP‐mock. This proliferation was suppressed by adding human IL13 recombinant protein to both LNCaP‐IL13Rα2 and LNCaP‐mock cells (Figure 2C). However, in androgen‐depleted conditions, the proliferation of LNCaP‐IL13Rα2 was higher than LNCaP‐mock. Moreover, adding human IL13 recombinant protein (25 ng/ml) enhanced the proliferation of LNCaP‐IL13Rα2 cells (Figure 2D). These results indicated that IL13Rα2 overexpression‐induced LNCaP cell proliferation and blocked the anti‐proliferative effect of IL13 only in androgen‐depleted conditions.

### 3.3 IL13Rα2 overexpression enhanced tumor formation and castration‐resistant growth in vivo

**FIGURE 3:**
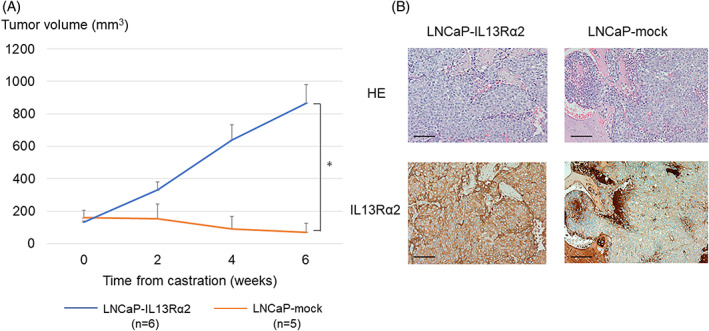
Tumor growth of IL13Rα2‐overexpressing LNCaP cells in castrated mice. A. The mice bearing LNCaP‐IL13Rα2 (*n* = 6) and LNCaP‐mock (*n* = 5) were castrated when the tumor volume reached 200 mm^3^. Sequential changes in the volumes of tumors after the castration of mice. B. Hematoxylin and eosin (HE) staining and IL13Rα2 immunohistochemistry of CDX tumors of LNCaP‐IL13Rα2 and LNCaPmock cells (Scale bars: 100 μm)

LNCaP‐mock and LNCaP‐IL13Rα2 were subcutaneously injected into both sides of five nude mice each, and tumor volumes were measured once a week. Five weeks after inoculation, tumors were detected in five of 10 (50%) sites in LNCaP‐mock cells and nine of 10 (90%) sites in LNCaP‐IL13Rα2 cells. All mice were castrated when the tumor volume reached 200 mm^3^; the tumor growth of LNCaP‐mock cells was significantly suppressed by the castration of mice compared with that of LNCaP‐IL13Rα2 cells (Figure 3A). These results indicated that the overexpression of IL13Rα2 enhanced the tumorigenic potential as well as castration‐resistant proliferation of LNCaP cells. The expression of IL13Rα2 in the tumor cells could be detected by the immunohistochemical analyses using anti‐IL13Rα2 antibody (Figure 3B).

### 3.4 IL13Rα2 expression was higher in the prostate cancer cells of patients acquiring castration resistance

**FIGURE 4:**
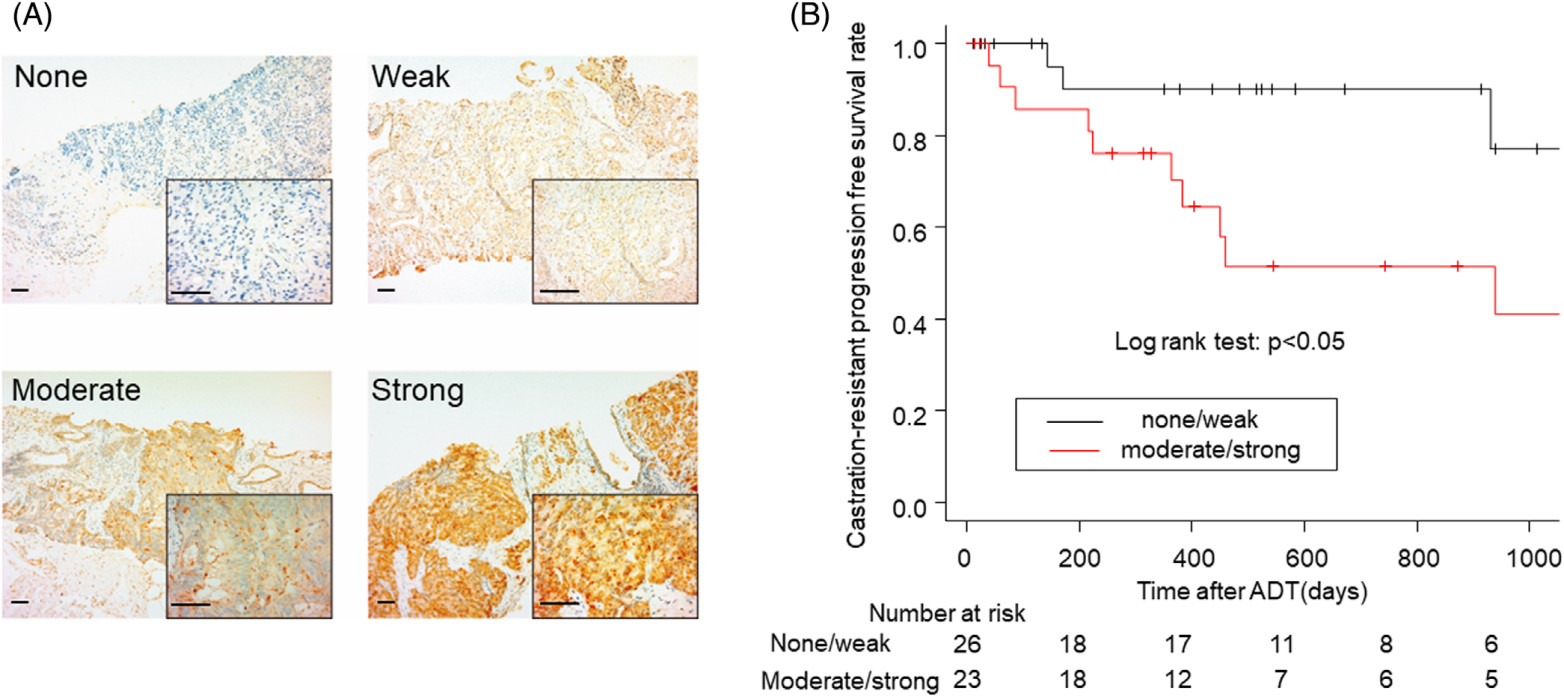
IL13Rα2 expression levels in prostate cancer biopsy samples and their association with time to castration‐resistance. A. IL13Rα2 expression levels (none, weak, moderate, and strong) in prostate cancer tissues obtained at diagnosis of prostate cancer before patients had undergone any treatment (with 40X and 400X magnification, Scale bars: 100 μm). B. Kaplan–Meier curves of castration‐resistant progression‐free survival of patients with none/weak (*n* = 26) and moderate/strong (*n* = 23) expression of IL13Rα2. *p* < 0.05 in log rank test

Immunohistochemical analyses of IL13Rα2 were performed in prostate cancer tissues obtained at diagnosis of prostate cancer before patients had undergone any treatment. The expression levels of IL13Rα2 were evaluated by a urological pathologist (S.M.). Immunoreactive staining intensity was judged by the percentage of cancer cells in which the cancer cell membranes were stained with or without cytoplasmic staining (e.g., if 80 out of 100 cells were stained, staining was 80%). Then, the expression levels were classified as follows: strong, >81%; moderate, 31%–80%; weak, 2%–30%; and none, <1% (Figure 4A). Among 49 patients, 26 were classified as none or weak and 23 were classified as moderate or strong. Between the groups with none/weak and moderate/strong IL13Rα2 expression, serum PSA levels and Gleason scores were not significantly different. However, the rate of patients with distant metastasis was significantly higher in the group with moderate/strong IL13Rα2 expression than the group with none/weak IL13Rα2 expression (65% and 31%, *p* = 0.02) (Table 1). The patients with none/weak IL13Rα2 expression had a longer time to castration‐resistant progression than those with moderate/strong IL13Rα2 expression (*p* < 0.05) (Figure 4B). These results indicated that the expression levels of IL13Rα2 in prostate cancer tissues could be a tissue biomarker to predict castration resistance.

**TABLE 1:** Patient characteristics in the groups of IL13Ra2 staining levels

IL13Ra2 staining levels	None~weak	Moderate~strong	
Number (*n*)	26	23	
Median (±SD) age (y)	76.0 (±6.84)	78.1 (±9.13)	*P* = 0.37
Median (±SD) serum PSA (ng/ml)	334 (±895)	1151 (±2299)	*P* = 0.10
*Gleason score*			
7 or less	8 (31%)	2 (9%)	
8 or higher	18 (69%)	21 (91%)	*P* = 0.08
*Distant metastasis*			
No	18 (69%)	8 (35%)	
Yes	8 (31%)	15 (65%)	*P* = 0.02

### 3.5 Serum IL13Rα2 concentration was higher in patients with castration‐resistant prostate cancer

**FIGURE 5:**
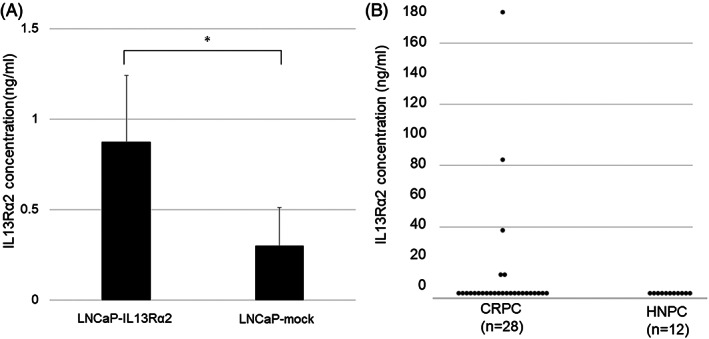
IL13Rα2 concentration in supernatant of cell lines and serum samples of prostate cancer patients. A. IL13Rα2 concentrations measured by ELISA in the supernatant of LNCaP‐IL13Rα2 and LNCaP‐mock cells (*p < 0.05). B. IL13Rα2 concentrations in serum samples obtained from patients with CRPC (*n* = 28) and HNPC (*n* = 12)

To establish methods to measure the serum concentration of IL13Rα2 in prostate cancer patients, ELISA was performed to determine the amount of IL13Rα2 protein secreted by cell lines. On the basis of the standard curve, the IL13Rα2 concentrations in the supernatants contained 80% confluent LNCaP‐mock and LNCaP‐IL13Rα2 cells incubated for 48 h (Figure 5A). This was significantly higher in LNCaP‐IL13Rα2 than in LNCaP‐mock, indicating that IL13Rα2 protein secreted by the prostate cancer cells could be measured by ELISA. IL13Rα2 levels were then measured in human serum samples of 12 HNPC patients and 28 CRPC patients using the same method. Serum IL13Rα2 levels could be measured in only five CRPC patients (18%) and in no HNPC patients (0%) (Figure 5B). These results indicated that the serum concentration of IL13Rα2 tended to be higher in some CRPC patients.

## 4 DISCUSSION

The standard treatment strategy for metastatic prostate cancer has shifted to the use of life‐extending therapies during the earlier stages of the disease. Primary docetaxel and ARATs, including abiraterone, enzalutamide, and apalutamide, in combination with ADT prolonged patient survival.^3^, ^4^, ^5^, ^6^ Upfront docetaxel was reported to be effective for patients with high‐volume metastasis but not for those with low‐volume metastasis.^23^ Upfront abiraterone was reported to significantly prolong the survival of patients with high‐risk prostate cancer with high Gleason scores, multiple bone metastases, or visceral metastasis.^4^ These results suggested that the metastatic volume might be a biomarker for selecting patients for upfront therapies. However, other ARATs were reported to be effective even for patients with low‐volume metastasis. To identify patients who would benefit from upfront therapies, novel biomarkers for predicting the efficacy of ADT for mHNPC patients are needed.

PDX models are characterized by the direct engraftment of patient‐derived tumor tissues into immunocompromised mice. PDXs can maintain the original histology, as well as the molecular and genetic characteristics of the source tumor.^24^ In the field of prostate cancer research, the most important characteristics are the efficacy of hormone therapy. The limited availability of human prostate cancer cell lines that harbor both intact AR expression and androgen dependency have slowed prostate cancer research. In the intact male mouse, the androgen‐dependent cells have a proliferative advantage and eventually develop into androgen‐dependent PDXs. These tumors shrink after the castration of mice, exhibiting castration‐sensitivity. However, in some PDX models, the tumor growth is not suppressed by the castration of mice, exhibiting castration resistance. We previously established several PDX models that harbor various sensitivity levels to castration.^7^, ^8^, ^9^ In this study, gene expression patterns were compared between castration‐sensitive and castration‐resistant PDX models to explore the genes associated with their castration sensitivity.

IL‐13 is associated with cellular immunity to tumor formation.^25^ IL‐13 is a cytokine secreted by T helper type 2 cells, CD4 cells, natural killer T cell, mast cells, basophils, eosinophils and neurocytes. It is a mediator of allergic inflammation and different diseases including asthma.^26^ IL‐13 activates the IL‐13 receptor complex to elicit signaling of the JAK1/STAT6 pathway, which may promote cell differentiation and inhibit tumor growth.^27^ The biological function of IL13Rα2 is less clear. Whereas it has been considered a decoy receptor,^10^ recent studies suggest that IL13Rα2 may have signaling potential.^28^ High‐level expression of this receptor in tumor cells would result in a situation in which the anti‐tumor activity of IL‐13 is compromised, leading to the nullification of cellular immunity and the occurrence of tumor escape. Therefore, IL13Rα2 has been shown to be a promising target for cancer treatment including prostate cancer.^29^ It was reported that exogenous IL13Rα2 expression rendered prostate cancer cells sensitive to the cytotoxic therapy.^30^ Moreover, IL13Rα2 protein was highly expressed in aggressive and metastatic prostate cancer cells and antagonized IL‐13 function.^14^ In our study, IL13Rα2 was highly expressed in castration‐resistant PDX tumors compared with castration‐sensitive tumors, indicating that the IL13Rα2 has a potential to predict the castration‐sensitivity of prostate cancer. However, the expression of IL13Rα2 did not change after the castration of mice in the gene expression profile of pre‐ and post‐castration tumor tissue of KUCaP2,^8^ indicating that the IL13Rα2 increase during the castration therapy might not contribute to the acquisition of castration‐resistance of prostate cancer.

IL13Rα2 overexpression in LNCaP cells suppressed cell proliferation in normal conditions, even under the administration of IL‐13. However, IL13Rα2 overexpression enhanced cell proliferation in androgen‐depleted conditions. Moreover, cell proliferation was enhanced by IL‐13 administration, and IL13Rα2 overexpression enhanced castration‐resistant growth in vivo more significantly than in vitro. It also enhanced tumorigenic potential. On the basis of these results, it was suggested that the IL13Rα2 protein might protect prostate cancer cells specifically in a starved state under treatment with ADT. The overexpression of IL13Rα2 did not change the expression levels of AR and PSA (Supplemental Figure 2). These results indicated that the IL13Rα2 was associated with in vitro and in vivo castration‐resistant cell proliferation of prostate cancer not correlating with AR activation.

IL13Rα2 is a membranous protein and its expression levels in prostate cancer tissues can be evaluated by immunohistochemical analyses. We found that the prostate cancer patients with higher expression of IL13Rα2 in biopsy tissues had a significantly shorter time to castration resistance after ADT. These results indicated that IL13Rα2 could be a potential tissue biomarker to predict the castration resistance of prostate cancer. To evaluate the utility of IL13Rα2 as a biomarker for selecting appropriate patients in which to use upfront docetaxel or ARATs, we need to examine the efficacy of docetaxel or ARATs for these patients with higher expression of IL13Rα2. Because IL13Rα2 is considered to be a decoy receptor,^10^ it might be secreted from cancer cells into serum. IL13Rα2 protein concentration could be measured by ELISA in the supernatant of IL13Rα2‐overexpressing LNCaP. Using the same methods, IL13Rα2 concentrations in serum samples of prostate cancer patients were measured. The serum levels of IL13Rα2 tended to be higher in some CRPC patients compared with HNPC patients. However, the serum levels of IL13Rα2 were low in the majority of CRPC patients. The castration resistance of prostate cancer is caused by multiple factors. Our study indicated that IL13Rα2 might be one of the mechanisms associating with castration resistance. These results indicated that IL13Rα2 might be a potential serum marker in CRPC patients.

This study has several limitations. All the mice harboring KUCaP9 died after the RNA sequence and the strain disappeared. Therefore, tumor tissue of KUCaP9 could not obtained for evaluating the expression levels of IL13Rα2 mRNA and protein. The expression levels of IL13Rα2 mRNA in KUCaP2 were lower than those in KUCaP7. The expression levels of IL13Rα2 mRNA were higher in PC3 cells than in LNCaP cells. However, IL13Rα2 protein in PC3 cells could not be detected by western blotting using IL13Rα2 antibody. Endogenous IL13Rα2 protein was detected only in KUCaP7 and human testis tissue (Supplemental Figure 1). These results were consistent with the previous report.^14^ Therefore, knockdown of endogenous *IL13RA2* could not be performed. IL13Rα2 was higher in castration‐resistant prostate cancer PDX tumor and clinical samples. The expression levels of IL13Rα2 did not change by the castration of mice. However, it was not elucidated whether the sequential changes of IL13Rα2 expression during the castration therapy was correlated with castration‐resistance of prostate cancer. IL13Rα2 concentration in the serum samples of some CRPC patients tended to be higher than that of HNPC patients. However, the IL13Rα2 expression levels in tissue samples of these patients were not evaluated in this study. Some of the prostate cancer cells expressing IL13Rα2 might be escape from the castration therapy leading to castration resistance. Therefore, the IL13Rα2 is not the sole parameter to be considered for castration resistance but an additional parameter. To evaluate the utility of serum biomarkers to predict the castration resistance of prostate cancer patients, a prospective study to examine the IL13Rα2 expression levels in tissue and serum samples of metastatic HNPC patients is needed.

## 5 CONCLUSIONS

In conclusion, IL13Rα2 was highly expressed in a castration‐resistant prostate cancer PDX model and was associated with the castration resistance of prostate cancer cells. Thus, it might be a potential biomarker for predicting the castration resistance of prostate cancer patients.

## AUTHOR CONTRIBUTIONS

**Masato Fujii:** Data curation (supporting); methodology (supporting); visualization (supporting). **Yasuhisa Nagata:** Investigation (supporting). **Kozue Nakahara:** Data curation (supporting). **Shoichiro Mukai:** Investigation (supporting); visualization (lead). **Kosuke Okasho:** Data curation (supporting); formal analysis (supporting); investigation (supporting); methodology (supporting). **Yuki Kamiyama:** Investigation (supporting); methodology (supporting). **Shusuke Akamatsu:** Investigation (supporting); methodology (supporting); supervision (supporting). **Takashi Kobayashi:** Supervision (supporting). **Kei Iida:** Data curation (supporting); formal analysis (supporting); methodology (supporting). **Masatsugu Denawa:** Data curation (supporting); formal analysis (supporting); validation (supporting). **Masatoshi Hagiwara:** Data curation (supporting); investigation (supporting); methodology (supporting). **Takahiro Inoue:** Supervision (supporting). **Osamu Ogawa:** Supervision (supporting). **Toshiyuki Kamoto:** Funding acquisition (lead); supervision (lead).

## FUNDING INFORMATION

Grants‐in‐aid for scientific research were awarded to the authors by the Ministry of Education, Culture, Sports, Science and Technology of Japan.

## CONFLICTS OF INTEREST

No potential conflicts of interest were disclosed.

## ERHICS STATEMENT

The atricle adhered to the principles outlined in the Declaration of Helsinki.

## Supporting information


**SUPPLEMENTAL TABLE 1** Expression levels of genes highly expressed in castration resistant PDX models (top 30)

**SUPPLEMENTAL FIGURE 1** IL13Rα2 mRNA and protein expression levels in KUCaPs and cell lines.
A. IL13RA2 mRNA expression (based on GAPDH) in KUCaP7, KUCaP2 and KUCaP10 tumors detected by real‐time PCR (**p < 0.005). B. IL13RA2 mRNA expression (based on GAPDH) in LNCaP and PC3 cells detected by real‐time PCR (**p < 0.005). C. Expression levels of IL13Rα2, AR, and PSA (based on α‐tubulin) in KUCaP7, KUCaP2, KUCaP10, testis (positive control of IL13Rα2), LNCaP and PC3 detected by western blotting.

**SUPPLEMENTAL FIGURE 2** AR and PSA mRNA expression levels in LNCaP overexpressing IL13Rα2.
AR and KLK3 mRNA expression (based on GAPDH) in LNCaP‐IL13Rα2 and LNCaP‐mock cells detected by real‐time PCR (*p < 0.005).

## Data Availability

Data sharing is not applicable to this article as no new data were created or analyzed in this study.
