# Supplementary material for: Identification of the α2 chain of interleukin‐13 receptor as a potential biomarker for predicting castration resistance of prostate cancer using patient‐derived xenograft models
Source: Cancer Rep (Hoboken). 2022 Aug 9;6(2):e1701. doi: 10.1002/cnr2.1701 (PMC9939991; doi:10.1002/cnr2.1701)
Supplement: Supplementary file 1 — SUPPLEMENTAL TABLE 1 Expression levels of genes highly expressed in castration resistant PDX models (top 30) [file CNR2-6-e1701-s003.doc]

| Supplement table 1.  Expression levels of genes highly expressed in castration resisntat PDX models (top 30) | | | | | |
| --- | --- | --- | --- | --- | --- |
| ID | Description | Average expression levels* | | Ratio CS/CR | p |
|  |  | CR(KUCaP 7 and 9) | CS(KUCaP 2 and 10) |  |  |
| NM_001097594 | *XAGE1B* | 4.90392 | 0.00000 | 0.00000 | 0.00000 |
| NM_001097597 | *XAGE1C* | 4.90392 | 0.00000 | 0.00000 | 0.00000 |
| NM_001097592 | *XAGE1A* | 4.90292 | 0.00000 | 0.00000 | 0.00000 |
| NM_001097604 | *XAGE1E* | 4.90292 | 0.00000 | 0.00000 | 0.00000 |
| NM_020411 | *XAGE1D* | 4.90292 | 0.00000 | 0.00000 | 0.00000 |
| NM_030774 | *OR51E2* | 6.48922 | 0.00000 | 0.00000 | 0.00002 |
| NM_004791 | *ITGBL1* | 2.96797 | 0.00000 | 0.00000 | 0.00003 |
| NM_199261 | *TPTE* | 1.43845 | 0.00000 | 0.00000 | 0.00011 |
| NM_000824 | *GLRB* | 2.05418 | 0.00000 | 0.00000 | 0.00038 |
| NM_013250 | *ZNF215* | 1.18412 | 0.00000 | 0.00000 | 0.00044 |
| NM_016608 | *ARMCX1* | 4.16750 | 0.00000 | 0.00000 | 0.00047 |
| NM_001098405 | *GAGE12F* | 4.58920 | 0.00305 | 0.00066 | 0.00019 |
| NM_001477 | *GAGE12I* | 4.91237 | 0.00348 | 0.00071 | 0.00003 |
| NM_001127199 | *GAGE12D* | 5.06213 | 0.00453 | 0.00090 | 0.00040 |
| NM_001098408 | *GAGE12C* | 5.10890 | 0.00472 | 0.00092 | 0.00039 |
| NM_001098418 | *GAGE12E* | 5.10890 | 0.00472 | 0.00092 | 0.00039 |
| NM_001098410 | *GAGE12H* | 5.09920 | 0.00472 | 0.00092 | 0.00039 |
| NM_001472 | *GAGE2C* | 4.29310 | 0.00435 | 0.00101 | 0.00027 |
| NM_001098409 | *GAGE12G* | 4.99245 | 0.00662 | 0.00133 | 0.00019 |
| NM_000640 | *IL13RA2* | 2.92097 | 0.00603 | 0.00207 | 0.00028 |
| NM_152430 | *OR51E1* | 4.91728 | 0.01278 | 0.00260 | 0.00033 |
| NM_012307 | *EPB41L3* | 2.35447 | 0.00800 | 0.00340 | 0.00012 |
| NM_001171020 | *MUM1L1* | 2.43227 | 0.01532 | 0.00630 | 0.00047 |
| NM_014491 | *FOXP2* | 1.92900 | 0.01237 | 0.00641 | 0.00034 |
| NM_024677 | *NSUN7* | 2.55255 | 0.02998 | 0.01175 | 0.00045 |
| NM_001145107 | *NELL2* | 2.80038 | 0.04882 | 0.01743 | 0.00005 |
| NM_178540 | *C1QTNF9* | 0.19090 | 0.00358 | 0.01877 | 0.00000 |
| NM_144586 | *LYPD1* | 1.26515 | 0.03483 | 0.02753 | 0.00005 |
| NM_001079821 | *NLRP3* | 1.79527 | 0.05057 | 0.02817 | 0.00019 |
| NM_052959 | *PANX3* | 1.45600 | 0.05508 | 0.03783 | 0.00041 |
| *logarithm transferred expression values(base=2), CR: castration resistant, CS: castration-sensitive | | | | | |
