# Supplementary figures and images for: Identification of the α2 chain of interleukin‐13 receptor as a potential biomarker for predicting castration resistance of prostate cancer using patient‐derived xenograft models
Source: Cancer Rep (Hoboken). 2022 Aug 9;6(2):e1701. doi: 10.1002/cnr2.1701 (PMC9939991; doi:10.1002/cnr2.1701)

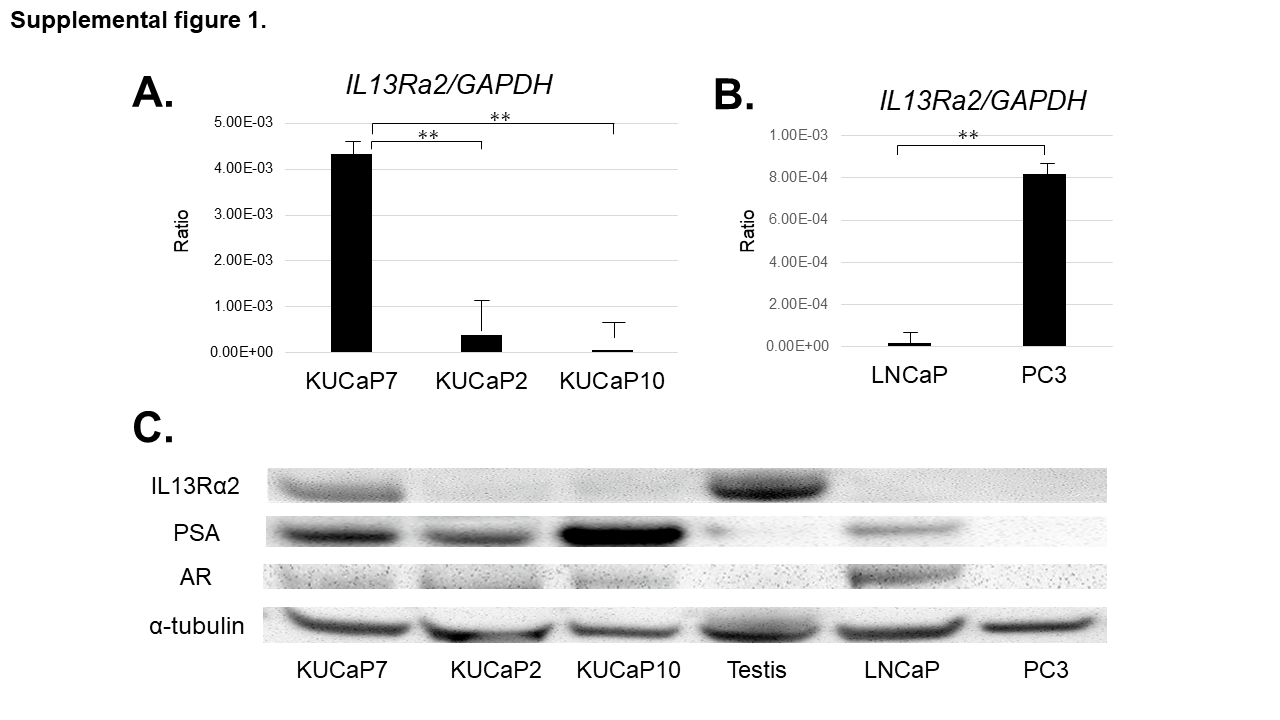

Supplement: Supplementary file 2 — SUPPLEMENTAL FIGURE 1 IL13Rα2 mRNA and protein expression levels in KUCaPs and cell lines. A. IL13RA2 mRNA expression (based on GAPDH) in KUCaP7, KUCaP2 and KUCaP10 tumors detected by real‐time PCR (**p < 0.005). B. IL13RA2 mRNA expression (based on GAPDH) in LNCaP and PC3 cells detected by real‐time PCR (**p < 0.005). C. Expression levels of IL13Rα2, AR, and PSA (based on α‐tubulin) in KUCaP7, KUCaP2, KUCaP10, testis (positive control of IL13Rα2), LNCaP and PC3 detected by western blotting. [file CNR2-6-e1701-s002.tif]

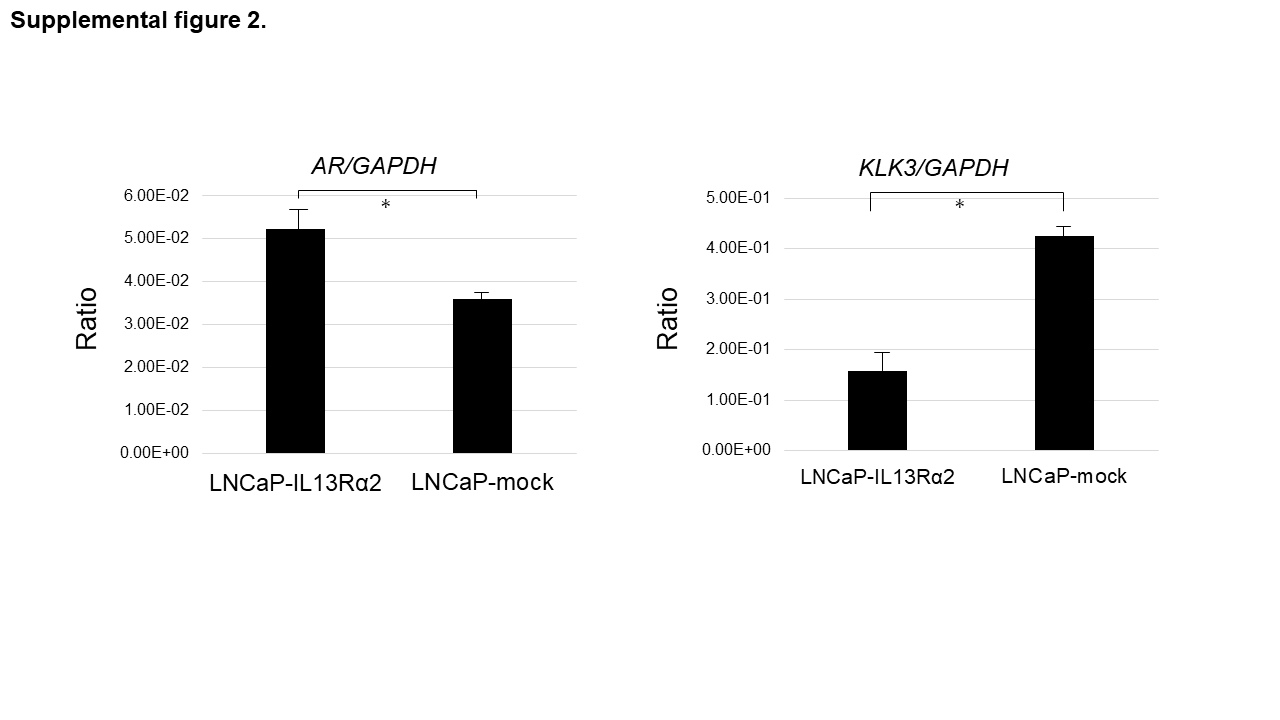

Supplement: Supplementary file 3 — SUPPLEMENTAL FIGURE 2 AR and PSA mRNA expression levels in LNCaP overexpressing IL13Rα2. AR and KLK3 mRNA expression (based on GAPDH) in LNCaP‐IL13Rα2 and LNCaP‐mock cells detected by real‐time PCR (*p < 0.005). [file CNR2-6-e1701-s001.tif]
